# Liposarcoma in the Inguinal Canal: Challenges in Preoperative Diagnosis and Importance of Routine Pathological Examination of “Hernia Sacs”

**DOI:** 10.1155/2018/5929626

**Published:** 2018-09-06

**Authors:** Christopher A. Febres-Aldana, Jin Min, Marc Rafols, Irvin Willis, John Alexis

**Affiliations:** ^1^Arkadi M. Rywlin MD, Department of Pathology and Laboratory Medicine, Mount Sinai Medical Center, Miami Beach, FL, USA; ^2^Nova Southeastern University, K.C. Patel College of Osteopathic Medicine, Davie, FL, USA; ^3^Department of Surgery, Mount Sinai Medical Center, Miami Beach, FL, USA

## Abstract

Liposarcoma is the most common histologic subtype of soft tissue sarcoma in the retroperitoneum. The distinction of primary cord liposarcomas, which arise in and are confined to the inguinal canal, from inguinoscrotal extension of a retroperitoneal tumor is mandatory. Both can be found incidentally in inguinal hernia sac specimens. Preoperative diagnosis is essential for adequate surgery with clear margins. We present a clinicopathological correlation of two men with slowly growing right para-testicular masses diagnosed as inguinal hernias. Pathological examination revealed well-differentiated lipoma-like liposarcoma and well-differentiated liposarcoma mixed type (lipoma-like and sclerosing types), respectively. The first tumor was considered a primary cord liposarcoma with no recurrence on follow-up. The second tumor showed an unusual growth pattern of discontinuous nodules that gave the false impression of complete resection. This growth pattern may explain why inguinal liposarcomas have a high recurrence rate despite apparently negative surgical margins. A follow-up CT scan exposed a fatty tumor in the retroperitoneum of the second patient. Careful interpretation of imaging studies in patients with fatty inguinal masses is mandatory to rule out a retroperitoneal or intraperitoneal component. Although the two cases herein discussed represent less than 0.1% of the total inguinal hernia sacs examined over the past five years in our pathology department, we recommend routine examination of all “mass-containing” hernia sacs as missing the diagnosis of liposarcoma can lead to substantial morbidity and mortality.

## 1. Introduction

Liposarcomas are malignant soft tissue neoplasms that can show adipocytic differentiation and represent up to 50 percent of the estimated 15,000 soft tissue sarcomas diagnosed each year in the United States. Most occur in middle-aged to elderly adults with similar incidence in both genders [1]. The prognosis depends on the anatomic site, grade, and resectability. For instance, the five-year survival rate in dedifferentiated liposarcomas and other high-grade liposarcomas (most common in retroperitoneum) is 18%, while that of low-grade well-differentiated type/atypical lipomatous tumor (most common in extremities) is 85%, these figures remaining unchanged over the past 40 years [2]. The high mortality rate in most cases is due to difficulty achieving full resection and late detection. Liposarcomas can manifest anywhere in the body, and the vast majority arise in the extremities followed by the retroperitoneum. There are few reports of liposarcomas arising in the inguinal canal or spermatic cord, some of them representing a direct extension from an intra-abdominal location [3, 4]. Herein, we describe two cases of liposarcomas presenting as fat-containing inguinal hernia sacs and diagnosed after the pathological examination.

## 2. Case Presentation

### 2.1. Case 1

**Figure 1:**
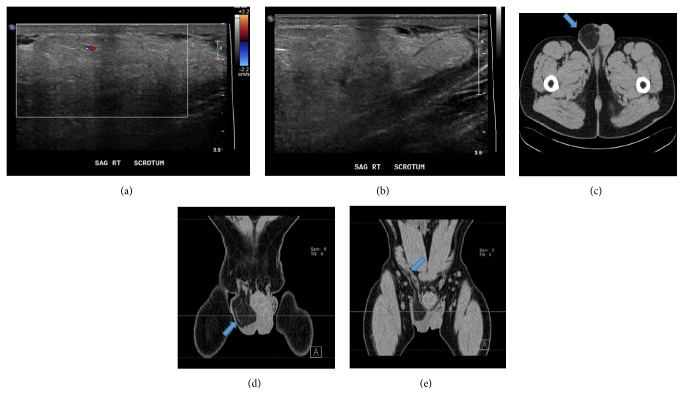
Doppler ultrasound in case 1 revealed a hyperechoic, solid, and heterogeneous lesion with minimal flow (a), extending to the scrotum (b). CT imaging in case 2 showed a low density mass with septations in the right scrotal sac ((c), axial view). The bulky mass was compressing the testicle ((d), coronal view) and showed spread into the abdominal cavity through the inguinal canal ((e), coronal view, arrow).

**Figure 2:**
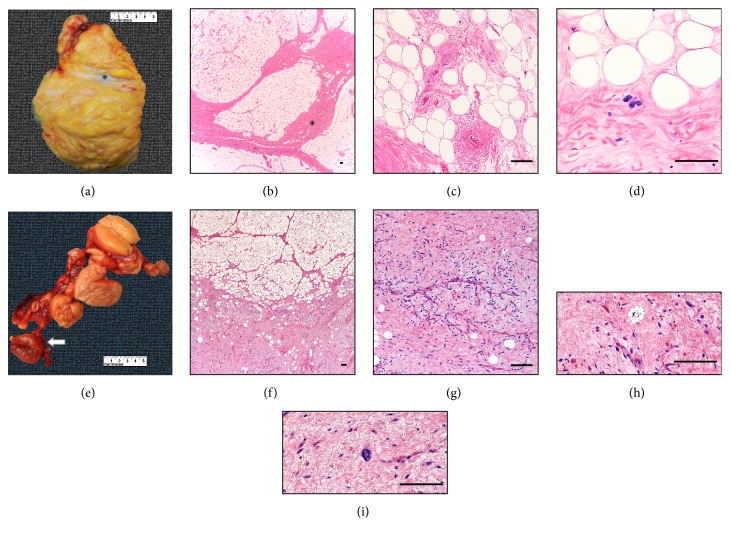
Gross and microscopic tumor examination in case 1 (a-d) and case 2 (e-i). See text for further explanation. Bar= 100 *μ*m.

A 78-year-old man presented with a slowly growing, painless, immobile right hemiscrotal mass over a nine-month period. An ultrasound study revealed a large right inguinal hernia containing herniated intra-abdominal fat (Figures 1(a) and 1(b)). On surgical exploration, the mass was encasing the right testicle requiring radical orchiectomy for complete resection. Gross examination revealed an 11 × 5.5 cm mass composed of adipose tissue with a lobulated cut surface and thick fibrous septations (Figures 2(a) and 2(b)). The blood vessels exhibited thickened, collagenized walls (Figure 2(c)). There were scarce atypical, nonlipogenic spindle cells with enlarged, irregular, pleomorphic, and hyperchromatic nuclei within the fibrous tissue (Figure 2(d)). There was no necrosis, nor mitotic figures. These findings are diagnostic of well-differentiated lipoma-like liposarcoma, grade 1. The margins were involved; thus the patient received radiation therapy. In the follow-up period after resection, the patient was recently examined and found to be disease-free.

### 2.2. Case 2

A 49-year-old man presented with a painless, nontender, nonreducible, firm, immobile, slowly enlarging right hemiscrotal mass over a one-year period. CT imaging revealed a right inguinal hernia with intraperitoneal fat extending inferiorly into the scrotal sac (Figures 1(c)–1(e)). Subsequently, a 14 × 10.5 cm membranous sac was excised. The hernia sac contained at least nine ovoid, circumscribed, separate, lobulated masses ranging from 2 to 8 cm in size tracking along the spermatic cord. The color varied from light brown to red brown (in contrast to case 1 where the mass was yellow) (Figure 2(e)). Microscopically, the predominant component was mature adipose tissue. However, the dark red component showed fibrous tissue with myxoid areas and variable numbers of adipocytes with significant variations in size and shape (Figure 2(f), bottom). Arborizing capillaries, lipoblasts (vacuolated cells with hyperchromatic scalloped nuclei), and atypical, nonlipogenic spindle cells were found predominantly in the myxoid component (Figures 2(g)–2(i)). This tumor also lacked necrosis and mitotic figures. This tumor was diagnosed as well-differentiated liposarcoma mixed type, lipoma-like, and sclerosing type, grade 1. On follow-up, a PET-scan revealed a nonhypermetabolic fatty mass along the distal anterior aspect of the right psoas, which was considered a retroperitoneal component of the inguinal tumor.

## 3. Discussion

Liposarcomas in the inguinal canal are rare. They are usually discovered incidentally during inguinal hernia repair surgery. The incidence of inguinoscrotal extension of retroperitoneal liposarcomas was 3.6% in a series of 168 patients [5]. There are numerous case reports of liposarcomas arising in the spermatic cord with no intra-abdominal component, so-called primary cord/inguinoscrotal liposarcomas [3, 6–8]. However, many times a retroperitoneal component has not been ruled out, casting doubt on the site of origin. Cord liposarcomas represent a small portion of all abdominal liposarcomas in comparison with primary retroperitoneal tumors, 4% versus 93%. Nonetheless, when a liposarcoma is identified in the inguinal canal, the probability of being either a primary tumor or an extension from the retroperitoneum is the same [5]. This distinction is critical for management and determination of prognosis. In the Rhu J. et al. study, the overall survival did not statistically differ between primary cord liposarcomas and retroperitoneal liposarcomas with inguinal spreading; curiously all fatalities occurred in patients with retroperitoneal liposarcomas who underwent “hernia repair” and not oncologic resection as the initial operation [5].

Cord liposarcomas often present as a slowly enlarging, recurrent inguinal hernia with a predilection for the right side [7, 8]. Liposarcomas show a propensity to invade locally rather than metastasize; hence, the prognosis depends on completeness (or otherwise) of excision. These neoplasms are challenging to identify solely upon gross examination as the neoplasm blends with the surrounding adipose tissue, which it resembles. Therefore, the diagnosis of liposarcoma requires microscopic examination, and in many instances molecular testing including the determination of MDM2 status. Furthermore, pathological inspection of inguinal masses helps rule out other para-testicular and spermatic cord neoplasms and determines margin status. In our pathology department, the two cases herein discussed represent less than 0.1% of the total inguinal hernia sacs examined over the past five years (2 out of 302 specimens). Considering that at our institution all inguinal hernia sacs are examined microscopically, this shows the rarity of liposarcomas in the inguinal canal. Montgomery E. et al. reported a similar rate of incidental liposarcomas (2 out of 1736 specimens) [9]. On the other hand, Wang T. et al. did not report any liposarcomas in a series of 800 inguinal hernias but identified other malignant tumors (0.4%) [10]. Regular histologic examination has been suggested for large (>10 cm) fatty masses only [9]; however, we recommend that any mass-containing hernia sac should be examined microscopically to avoid misdiagnosis with lipoma. Liposarcomas as small as 3 cm have been reported [5].

Imaging of mass-containing inguinal hernias is critical for planning the surgical approach. Identification of well-differentiated liposarcomas can be a challenge because they are usually poorly demarcated from surrounding normal fat, homogenous, and low density. In contrast, high-grade liposarcomas present as solid, heterogeneous, high-density masses [5–7]. They may show continuous expansion rather than scatter seeding distribution. Cord liposarcomas may be interspersed with fat stranding and soft tissue segments suggesting malignancy, but this is seen more frequently in retroperitoneal liposarcomas. The presence of internal septations may also help differentiate from a lipoma. However, infarcted lipomas can have imaging features of malignant tumors. If a tumor is involving the scrotal sac, ultrasound imaging can rule out other para-testicular neoplasms or mass-like lesions such as varicocele, hydrocele, and chronic epididymitis [7]. In fatty soft tissue neoplasms, high-resolution ultrasound images will reveal a hyperechoic, solid, and heterogeneous lesion without distinct borders often surrounding the testicle. Overall, imaging techniques cannot reliably differentiate lipomas from liposarcomas. Surgeons must have a high suspicion of malignancy when the mass is heterogeneous; therefore the resection can be planned to remove as much tumor as possible. The margin status is one of the most critical variables that dictate further management and determines prognosis [2].

Surgical management involves radical high orchiectomy with margins as close to the inguinal canal as possible. As with primary testicular malignancies, the ideal approach is via an inguinal incision as opposed to a trans-scrotal approach. Retroperitoneal lymph node resection is not recommended. There is still debate about the effectiveness of radiotherapy in the management of liposarcomas. Radio/chemotherapy has often been added as an adjuvant therapy to high-grade liposarcomas, but there is no benefit in overall mortality or recurrence rates [1, 2]. As the vast majority of liposarcomas originate in the retroperitoneum, there is the question whether the tumor originated in the inguinal canal or from another primary location. The tumor in case 1 seemed to be a cord liposarcoma; imaging before surgery did not show an intra-abdominal component. In case 2, a retroperitoneal fatty tumor was discovered after primary excision despite not being detected on preoperative imaging. The retrospective review of a CT scan did show a small fatty protrusion from the inguinal canal into the abdominal cavity (Figure 1(e)). In this case, the margin of resection was considered tumor free. However, this was a false negative margin and imaging result because this neoplasm demonstrated a discontinuous lobulated growth (separate nodules). This growth pattern may explain why inguinal liposarcomas have a high recurrence rate, up to 75%, in the prior surgical site, despite excision margins reported as tumor free [3].

In summary, tumors in the inguinal canal should be managed with caution. Liposarcomas involving the inguinal canal are rare but should be considered in the differential diagnosis. Further imaging workup is mandatory to rule out a retroperitoneal or intraperitoneal component. A preoperative distinction between cord liposarcomas and retroperitoneal liposarcomas with inguinoscrotal extension is relevant for adequate surgical resection and accurate determination of the margin status. The current treatment of choice is* en bloc* resection with radical orchiectomy and close follow-up with imaging. Lymph node dissection and radio/chemotherapy have not been shown to improve mortality.
